# Arctic lichen *Cladonia borealis*-induced cell death is mediated by p53-independent activation of Caspase-9 and PARP-1 signaling in human colorectal cancer cell lines

**DOI:** 10.71150/jm.2412012

**Published:** 2025-04-29

**Authors:** Ju-Mi Hong, Seul Ki Min, Kyung Hee Kim, Se Jong Han, Joung Han Yim, Sojin Kim, Youn-Jung Kim, Il-Chan Kim

**Affiliations:** 1 Division of Polar Life Sciences, Korea Polar Research Institute, Incheon 21990, Republic of Korea; 2 Department of Marine Sciences, Incheon National University, Incheon 22012, Republic of Korea; 3 Life Science Institute, Daewoong Pharmaceuticals, Youngin 17028, Republic of Korea; 4 Department of Polar Sciences, University of Science and Technology, Incheon 21990, Republic of Korea; 5 Department of Biomedical Laboratory Science, Daegu Health College, Daegu 41453, Republic of Korea

**Keywords:** apoptosis, arctic lichen *Cladonia borealis*, cancer, human colon carcinoma

## Abstract

The anti-cancer effects of *Cladonia borealis* (an Arctic lichen) methanol extract (CBME) on human colon carcinoma HCT116 cells were investigated for the first time. The proliferation of the HCT116 cells treated with CBME significantly decreased in a dose- and time-dependent manner. Flow cytometry results indicated that treatment with CBME resulted in significant apoptosis in the HCT116 cells. Furthermore, immunoblotting and qRT-PCR results revealed the expression of apoptosis-related marker genes and indicated a significant downregulation of the apoptosis regulator B-cell lymphoma expression and upregulation of the cleaved form of poly (ADP-ribose) polymerase as DNA repair and apoptosis regulators and central tumor suppressor p53. Therefore, CBME significantly inhibited cell proliferation by inducing apoptosis via the mitochondrial apoptotic pathway in colon carcinoma cells. Collectively, these data suggested that CBME contained one or more compounds with anti-cancer effects and could be a potential therapeutic agent. Further studies are required to identify candidate compounds and understand the mechanism of action of CBME.

## Introduction

Cancer is a disease with a high mortality rate characterized by the uncontrolled proliferation of abnormal cells and metastasis to all bodily organs or tissues and is difficult to treat at an advanced stage (Shahnaz et al., 2019). Colorectal cancer is the third most common malignancy worldwide, with more than 1.9 million cases diagnosed in 2022. Moreover, it is the second most common cause of cancer-related deaths (Lei et al., 2020). Surgery and chemotherapy are currently used to treat colorectal cancer; however, side effects, such as resistance to anti-cancer drugs and decreased immunity, have been observed. This has prompted the investigation of various natural extracts that are harmless to the human body for cancer prevention and treatment (Kawk et al., 2019).

Lichens are estimated to comprise 18,500 species worldwide and are robust organisms that can survive in extreme environmental conditions, such as the equator, South Pole, North Pole, and Highlands (Øvstedal and Lewis Smith, 2011). Lichens have long been used in numerous practical applications, including as a source of medicine (Gautam et al., 2021). The secondary metabolites produced by lichens are extremely diverse and unique substances that are difficult to find in other organisms. Studies have reported that these metabolites exhibit significant biological and pharmacological effects, such as anti-cancer, anti-oxidant, anti-bacterial, anti-inflammatory, and anti-viral effects (Basile et al., 2015).

Commonly known as the boreal cup lichen, *Cladonia borealis* (CB) belongs to the family Cladoniaceae. *Cladonia* sp. are found in polar, subpolar, and alpine regions, such as Alaska, the Antarctic Peninsula, the South Shetland Islands, and South Georgia. Several species of Cladonia numerous pharmacological properties, including anti-microbial, anti-oxidant, and anti-proliferative activities, as observed in in vitro experiments (Coskun et al., 2015; Ranković et al., 2011; Studzińska-Sroka et al., 2015). Certain studies have investigated the phylogenetic and genetic analyses of CB regarding its adaptation to the polar environment; however, the biological activity of CB in carcinogenesis and cancer therapy has not been comprehensively investigated (Cho et al., 2024; Park et al., 2012). In this study, we investigated the anti-cancer effects of CB methanol extract (CBME) on the human colorectal cancer cell line HCT116. To the best of our knowledge, this is the first study to report the effects of CBME on the prevention and treatment of colorectal cancer. The results of this study could be used to support CBME as a potential therapeutic agent.

## Materials and Methods

### Collection of the lichen and preparation of the extract

CB was collected around the Korea Arctic Research Station Dasan at NyAlesund, Svalbard, Norway (N78°91’51”, E11°90’44”) in July 2018. The freeze dried lichen CB (10 g) was crushed and used in powder form for extraction. Methanol was used as a solvent, and the lichen was soaked at room temperature and extracted three times (250 ml × 24 h). The extracted solvent was filtered and concentrated under reduced pressure using a rotary evaporator to obtain a crude methanol extract (21.7 mg). The nucleotide sequence of the genomic mitochondrial small subunit rDNA of the Arctic lichen CB was analyzed using BLAST for sequence similarity against the NCBI GenBank database. For in vitro studies, CBME was dissolved in dimethyl sulfoxide (DMSO, 20 mg/ml stock solution stored at -20℃) and diluted to the indicated concentrations.

### Cell culture

HCT116 cells were grown to confluence in Dulbecco’s Modified Eagle Medium (Sigma-Aldrich, USA) supplemented with 10% heat-inactivated fetal bovine serum (FBS, Invitrogen, Canada) and 1% (w/v) anti-biotic–anti-mycotic solution (Invitrogen, USA) in a humidified atmosphere with 95% air and 5% CO_2_ at 37℃.

### Cytotoxicity assay

Cell cytotoxicity was determined using the 3-(4,5-dimethyl-2-thiazolyl)-2,5-diphenyl-2H-tetrazolium bromide (MTT, USA) assay. HCT116 cells were seeded at a density of 1 × 10^5^ cells/ml in 96-well plates and incubated with various concentrations of CB for 24, 48, and 72 h. Subsequently, 5 μl of a 5 mg/ml MTT solution was added to the cells, followed by incubation at 37℃ for 4 h. Following treatment for 10 min with 100 μl of fresh DMSO to dissolve the crystals, the cells were detected under a microplate reader (Thermo Scientific Inc., USA), and absorbance was measured at 570 nm. Relative cell viability was calculated by comparing the absorbance with that of the untreated control group. All experiments were performed in triplicates.

### Morphological analysis

To investigate potential morphological changes, HCT116 cells were seeded at a density of 2.5 × 10^5^ cells/ml in 12-well plates and incubated for 24 h before treatment. After 24 h, cells were treated with the indicated concentrations of CB and treated with 1 µM doxorubicin (DOX), which served as the positive control for apoptotic morphology. Morphological changes in the HCT116 cells were observed using an inverted phase-contrast microscope (Olympus, Japan) following 24 h of treatment.

### Quantitative real-time polymerase chain reaction analysis

Total RNA was extracted, and RT-PCR was performed as previously described (Suh et al., 2018). For RT-PCR, single-stranded cDNA was synthesized from 1 μg of total RNA using oligo (dT) primers and M-MLV reverse transcriptase. The primer pairs used for each gene are listed in Table 1. RT-PCR was performed by monitoring the increase in the amount of SYBR Green in real time using a QuantStudio1 RT-PCR system (Thermo Fisher, USA). The RT-PCR thermal cycling conditions were as follows: 60 min at 37℃, 15 min cDNA synthesis at 72℃, 15 s denaturation at 95℃, 15 s annealing at 60℃, and 60 s elongation at 72℃ (for 40 cycles). β-Actin was used as a reference gene to normalize the expression level of each gene. RT-PCR data were collected using the QuantStudio1 detection system. Cycle threshold (Ct) values were determined using the automated threshold analysis, and primer quality was confirmed using a melting curve analysis. All experiments were performed in triplicate.

### Immunoblot analysis

Total cellular extracts were prepared according to our previous paper (Hong et al., 2015), separated by SDS-PAGE on an 8% gel, and separated proteins were transferred to a polyvinylidene fluoride (PVDF, Millipore Corporation, USA) membrane. The membrane was incubated with a blocking solution (5% skim milk) and then incubated with anti-PARP1, anti-Caspase-9, anti-cleaved caspase-9 and p53 antibodies overnight at 4°C. The following primary antibodies and dilutions were used: anti-PARP-1 (1:1000 dilution, Cell Signaling, USA), anti-Caspase-9 (1:1000 dilution, Cell Signaling), anti-cleaved caspase-9 (1:1000 dilution, Cell Signaling), anti-p53 (1:1000 dilution, Cell Signaling) and anti-actin (1:1000 dilution, Sigma Aldrich, USA). Membranes were washed three times with a Tris-buffered saline containing 0.1% Tween 20 (TBST), and then incubated with a 1:5000 dilution of horseradish peroxidase (HRP)-conjugated secondary antibody (Jackson Immune Research Inc., USA) for 1 h at 25°C. Membranes were washed three times with TBST, and then developed using an enhanced chemiluminescence (ECL) kit (Translab, Korea). For quantitative analysis, densitometric band values were determined using a Fusion Solo 6S Edge imaging system (Vilber, France).

### Apoptosis assays

Apoptosis and/or necrosis was investigated in the HCT116 cells using Annexin V/fluorescein isothiocyanate (FITC) and propidium iodide (PI) double staining. Briefly, 2 × 10^6^ cells/ml were seeded into 6-well plates. After 24 h, the cells were treated with lobaric acid and lobarstin at the indicated concentrations for 24 h. The cells were washed, harvested, and stained with Annexin V/FITC and PI (BD Biosciences, USA) according to the manufacturer’s instructions. Untreated cells stained with PI or Annexin V/FITC served as the controls. Samples were analyzed using a flow cytometer (Beckman Coulter Inc., USA).

### Wound-healing assays

For the wound-healing assays, HCT116 cell monolayers were seeded into 12-well dishes in a medium with 10% FBS and incubated for 24 h. A wound-healing assay kit (Abcam, UK) was used according to manufacturer’s instructions. After 24 h, the insert was carefully removed from the wells to begin the wound-healing assay, and the media were discarded. Subsequently, the cells were treated with CB at 0 (untreated), 25, and 50 μg/ml for 48 h, washed, and stained according the manufacturer’s instructions. Photographs were obtained using an inverted microscope at 48 h, and wound lengths were determined using ImageJ software.

### Statistical analysis

Values are expressed as the mean ± the standard error of the mean. The significance of the difference from the respective controls for each experimental test condition was assayed using Student’s t-test for each paired experiment. Differences were considered statistically significant at *P < 0.05 and **P < 0.01.

## Results

### Effect of CBME on cell viability

**Fig. 1.**
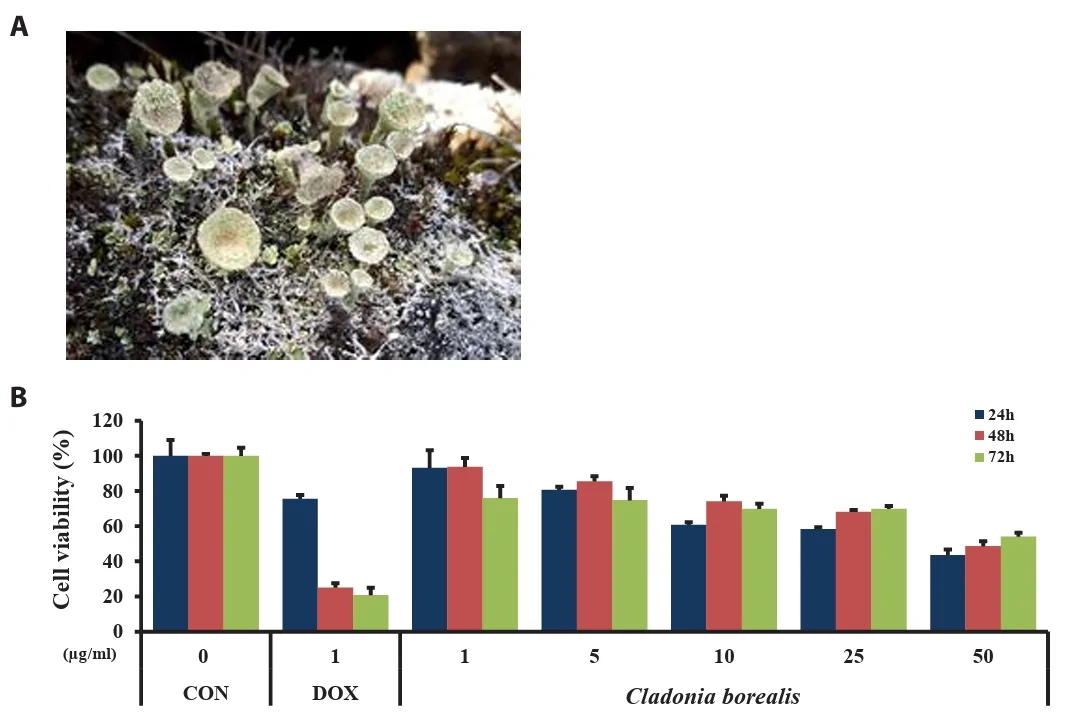
The Arctic lichen, *Cladonia borealis*, in its natural habitat (A). Effect of the methanolic extract of *Cladonia borealis* (CBME) on the proliferation of HCT116 cells (B). Cells were treated with various concentrations of CBME for 24, 48, and 72 h, and cell viability was measured using the MTT assay. Three independent experiments were performed, and the data are presented as the mean ± SEM.

**Fig. 2.**
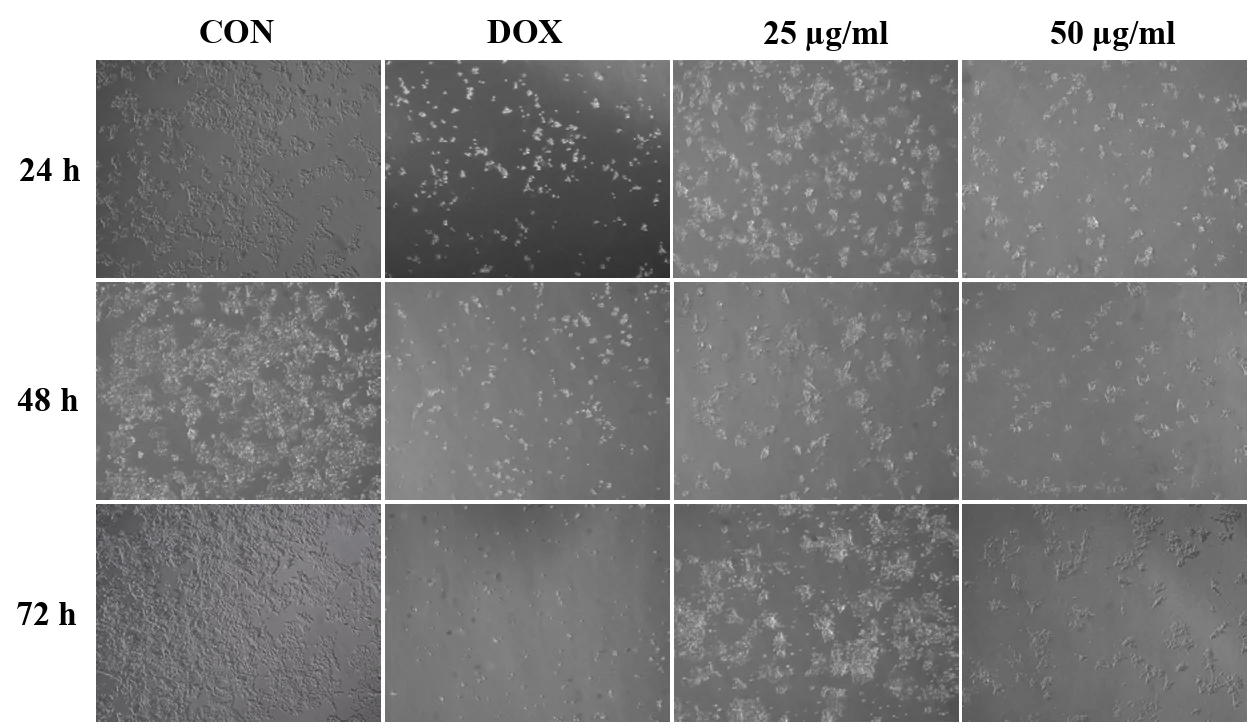
Morphological changes of HCT116 cells treated with CBME at 25 and 50 μg/ml for 24, 48, 72 h. Light microscopy photographs (40× objectives).

To investigate the effect of CBME on the viability of human colorectal cancer cells, HCT116 cells were treated with various concentrations of CBME (1, 5, 10, 25, and 50 μM) and DOX (1 μM, used as positive control) for 24, 48, and 72 h. The absorbance of HCT116 cells was measured using the MTT assay. As presented in Fig. 2, the viability of the cells in response to CBME treatment was significantly decreased in a dose- and time-dependent manner when compared with the untreated control (Fig. 1), with a half maximal inhibitory concentration (IC50) of 50 μg/ml in the HCT116 cell lines. These data suggested that CBME treatment inhibited the viability of human colorectal cancer cells.

### Effect of CBME on cell morphology

To investigate the effect of CBME on HCT116 cell morphology, the cells before and after treatment with CBME were observed under an optical microscope (Fig. 2). The untreated cells were uniformly distributed in the culture field and exhibited a polygonal shape. Following treatment with different concentrations (25 and 50 μM) of CBME for 24 h, the HCT116 cells transformed from polygonal to circular. Apoptosis causes significant changes in cell morphology and is typically accompanied by cell shrinkage, chromatin condensation, and rapid phagocytosis by neighboring macrophages. Furthermore, a significant reduction in cell number and an increased number of floating cells were observed in HCT116 cells.

### Effect of CBME on apoptosis-related mRNA and protein expression in HCT116 cells

**Fig. 3.**
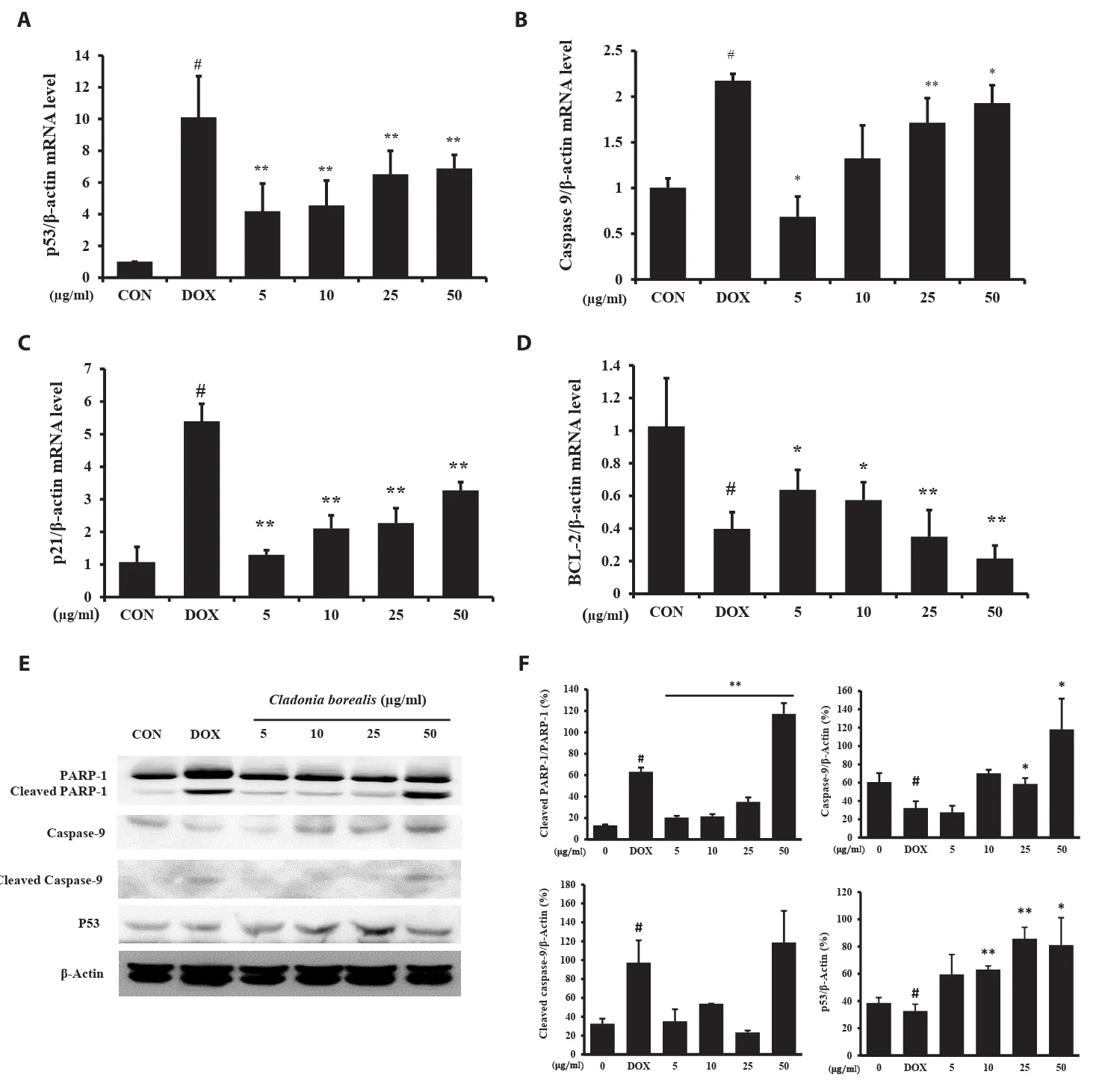
Effect of CBME on the apoptosis-related mRNA and protein gene levels in HCT116 cells. (A–D) mRNA levels of p53, Caspase-9, p21, and Bcl-2 analyzed using RT-qPCR. (E–F) Protein levels of PARP-1, cleaved PARP-1, Caspase-9, cleaved caspase-9, and p53 analyzed using western blotting. Data are presented as mean ± SD of three independent experiments (^*^p < 0.05, ^**^p < 0.01 versus control group).

To investigate whether apoptosis was caused by CBME, the expressions of the apoptosis-related p53, Caspase-9, p21, and Bcl-2 genes was measured using the primers listed in Table 1, and the protein expression of cleaved PARP-1 was confirmed. DOX-treated HCT116 cells exhibited changes in the expressions of p53, caspase-9, and p21, with a reduced expression of bcl-2. In HCT116 cells cultured for 24 h with CBME, a concentration-dependent increase in p53, capase-9, and p21 levels was observed, whereas that for bcl2 decreased (Fig. 3A–3D). The amount of the cleaved PARP-1, caspase-9 and p53 protein increased with 50 μg/ml CBME treatment compared to that of DOX-treated HCT116 cells (Fig. 3E and 3F). The increased expressions of the p53, caspase-9, and p21 genes and decreased expression of the bcl-2 gene promoted the cleavage of PARP-1, resulting in apoptosis.

### Effect of CBME on apoptosis in HCT116 cells

**Fig. 4.**
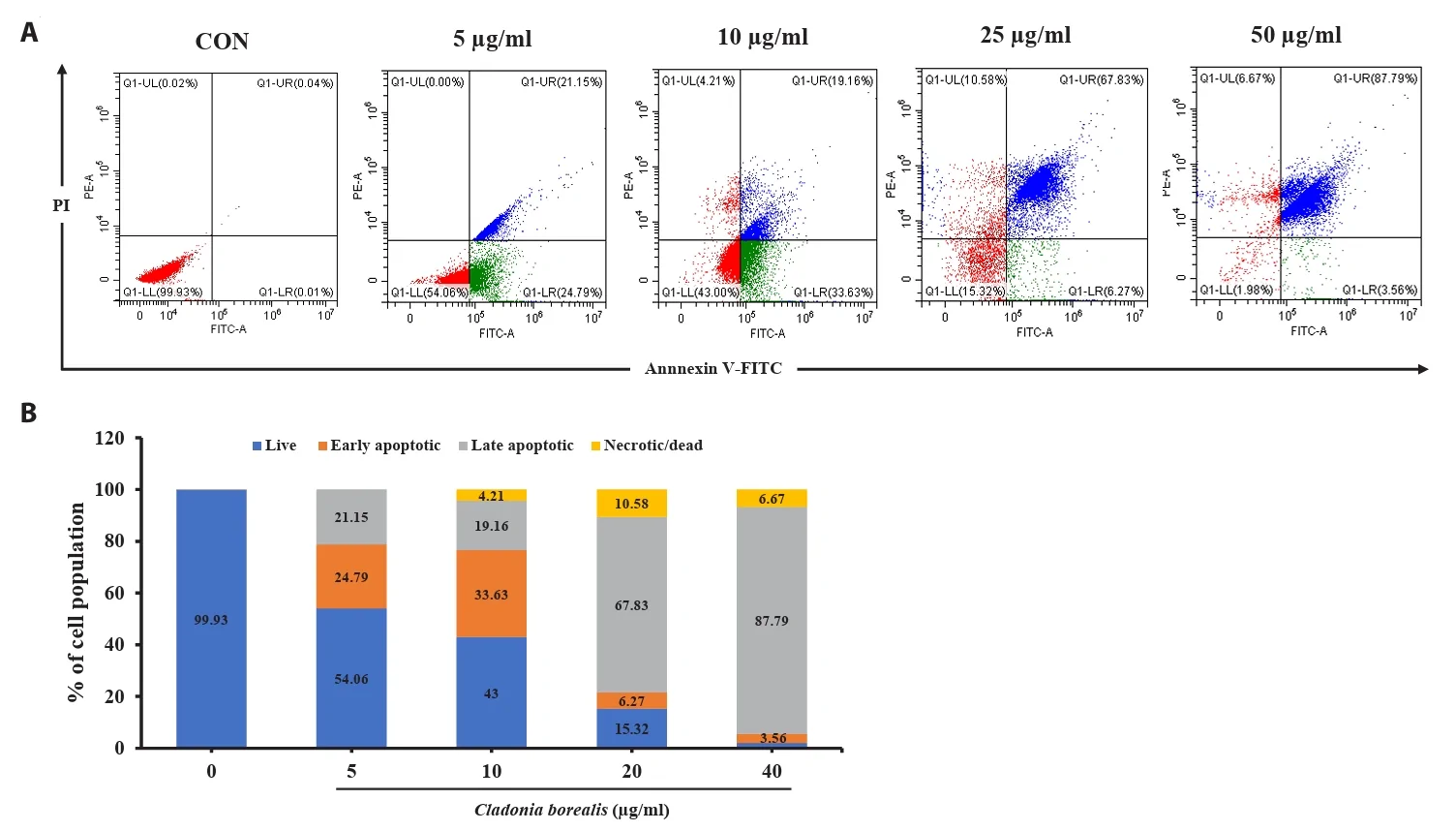
Effect of CBME on the apoptosis of HCT116 cells. (A) Cells were stained with Annexin V/propidium iodide (PI), and the apoptotic cell population was evaluated using flow cytometry. (B) Graphical representation of the percentages of live, early apoptotic, late apoptotic, and necrotic/dead cells.

We confirmed that the cytotoxic effect of CBME on cancer cells occurred via apoptosis; thus, we performed an Annexin V-staining-based flow cytometric analysis. As shown in Fig. 4, various concentrations of CBME-treated HCT116 cells significantly increased in both the early (Annexin V+/PI−) and late (Annexin V+/PI+) apoptotic cell populations. The apoptosis indices were 0.07%, 45.94%, 52.79%, 74.1%, and 91.35% at CBME concentrations of 0, 5, 10, 25, and 50 µg/ml, respectively. Early apoptosis occurred at a lower concentration (5 and 10 µg/ml) of CBME, whereas late apoptosis occurred at a higher concentration (20 and 40 µg/ml). These results suggested that CBME significantly induced apoptosis in the HCT116 cells in a concentration-dependent manner.

### Effect of CBME inhibits cell migration of HCT-116 cell monolayers

**Fig. 5.**
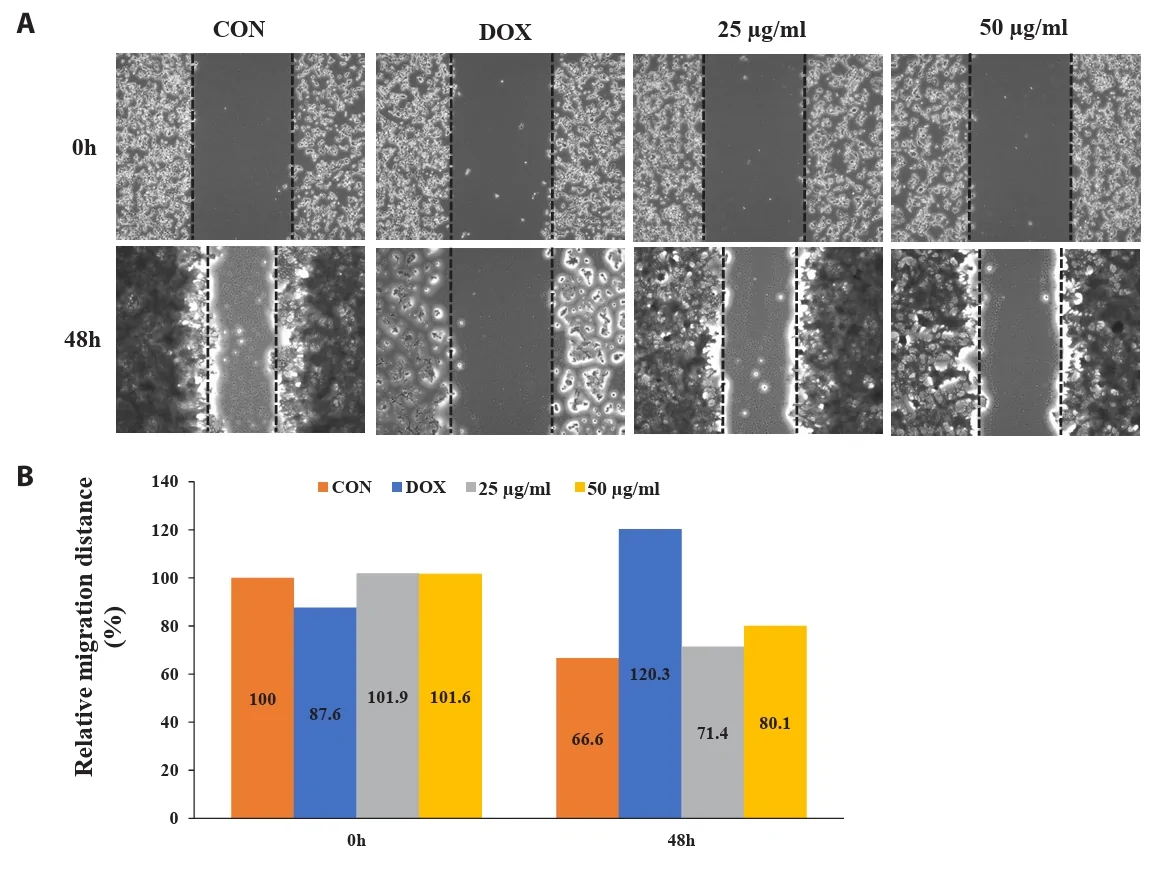
Fluorescent microscope images to evaluate wound healing in vitro in the scratch assay using a confluent monolayer of HCT116 cells. (A) Cell migration into the wound observed in response to an artificial injury. Gap closure was measured 48 h after initial scratch. (B) Quantification of relative migration distances for the wound-healing assay.

To determine the potential effect of CBME on cell migration, we performed a wound-healing assay. Figure 5 shows the images of the HCT116 cells at 0 and 48 h post-scratching and following treatment with DMSO (vehicle control), DOX (1 µM, positive control), and CBME (25 and 50 µg/ml). Cell migration inhibitions of 4.8% and 13.5% occurred in a concentration-dependent manner when 25 and 50 µg/ml of CBME treatment, respectively, were used for 48 h compared that that of the untreated samples. When 25 and 50 µg/ml of CB were compared with untreated (0) after treatment for 48 h, we found that inhibition of cell migration of 4.8% and 13.5%, respectively, occurred in a concentration-dependent manner.

## Discussion

Colorectal cancer is one of the most common human cancers and has one of the highest cancer-related mortality rates (Potter et al., 1993). The specific cause of colorectal cancer is unknown; however, it is linked to various factors, such as environmental factors, lifestyle changes, dietary changes, and genetic factors. In particular, a Western-style diet with high animal fat, high refined sugar, and low fiber contents plays an important role in colon cancer (Vernia et al., 2021). Surgery remains the only treatment for patients in the early stages of colorectal cancer; however, approximately 50% of patients relapse following surgery and often undergo metastasis, which may be due to failures in finding latent metastasis or in completely resecting the lesion (Su et al., 2015). Although postoperative radiotherapy and chemotherapy can extend the lifespan of patients by five years, their toxicity and side effects are of concern for improving the overall quality of life of patients (Oliphant et al., 2013). Therefore, safer and more effective treatments are required to improve the management of colorectal cancer.

DOX is an effective chemotherapeutic drug used for treating various carcinomas, including leukemia and bronchial, breast, stomach, and colon cancers (Yoneda and Cross, 2010). The chemotherapeutic effect of DOX proceeds via two mechanisms: intercalation into DNA and inhibition of topoisomerase II. These mechanisms alter the chromatin structure and promote the formation of active radicals, thereby damaging DNA, proteins, and cell membranes (Taymaz-Nikerel et al., 2018). However, the use of DOX in clinical practice is limited owing to its dose-dependent toxicity to the heart, brain, and kidneys (Prathumsap et al., 2020). To identify drugs that can improve the side effects of DOX, we investigated the pharmacological activities, including anti-biotic, anti-fungal, anti-oxidant, and anti-proliferative agents, of lichen extracts (Palacios-Moreno et al., 2019). Notably, these pharmacological properties have been reported for *Cladonia* species, but not for CB.

The MTT assay was first used to assess the effects of CBME on the growth and proliferation of colorectal cancer cells. CBME inhibited HCT116 cell viability in a dose- and time-dependent manner. In addition, the survival rate of the CBME-treated (10–50 μg/ml) HCT116 cells for 24 h was lower than that of the DOX-treated cells. To determine the mechanism of the anti-proliferation effects of CBME, we examined the effect of CBME on the apoptosis pathway. The results of the flow cytometric analysis indicated that apoptosis contributed to decreased cell viability following CBME treatment. Further studies have implied that CBME may induce apoptosis via the intrinsic mitochondrial pathway.

Based on our data, CBME significantly inhibited colorectal cancer cell growth and induced cellular apoptosis by modulating apoptotic genes, such as Caspase-9, Bcl-2, and p53. In general, apoptosis results in a comprehensive interaction among various apoptotic regulatory proteins, such as Caspase-9, Bcl-2, p53, and PARP-1 (Elmore, 2007). For example, Bcl-2 plays a crucial role in regulating physiological cell death and cell proliferation by inhibiting apoptosis and preventing the activation of pro-apoptotic caspase proteins such as caspase-9 (Avrutsky and Troy, 2021; Larrubia et al., 2013; Qian et al., 2022). Furthermore, p53 is a tumor suppressor gene that functions at the transcriptional level as a negative regulator of Bcl-2 and participates in cell cycle arrest and/or the induction of apoptosis (Ozaki and Nakagawara, 2011; Suh et al., 2017). PARP-1 is a stress-responsive protein that repairs DNA damaged by poly ADP-ribosylated nucleoproteins and regulates chromatin structure. The proteolytic cleavage of PARP-1 by caspase is an early indicator of apoptosis (Hong et al., 2018). We confirmed that CBME induced apoptosis by significantly downregulating Bcl-2 and upregulating cleaved caspase-9, p53, p21, and cleaved PARP-1 expressions.

In addition, flow cytometry was performed to determine whether the cytotoxic effects of CBME on the colorectcal cancer cells occurred through apoptosis. The population of apoptotic cells significantly increased in a dose-dependent manner following CBME treatment. Therefore, CBME significantly induced apoptosis in the HCT116 cells in a dose-dependent manner. In addition, the development of anti-cancer drugs that inhibit the migration of cancer cells is important because it can effectively inhibit the metastasis of cancer cells (An et al., 2020). CBME was confirmed to inhibit migration using a winding assay; thus, CBME could be used for developing anti-cancer drugs that suppress the metastasis of colon cancer cells.

Apoptosis causes changes in the cell morphology and results in cell death through a series of highly regulated biochemical events. As a typical sign of apoptosis, chromosomes condense in the nucleus and move toward the nuclear envelope, where DNA degradation is initiated (Sidhartha and Harihara, 2005). Plasma membrane blebbing is another feature of apoptosis. Phosphatidylserine, a lipid present only in the inner layer of the plasma membrane, becomes present in the outer layer. Nucleus and cytoplasmic fragments are degraded by apoptosis, and neighboring macrophages recognize and phagocytose and are subsequently degraded in the phagosomes (He et al., 2009). We observed that CBME caused cell shrinkage and changed cell shapes from polygonal to circular, with a significant decrease in cell number and an increased number of suspended cells.

In summary, our results demonstrated that CBME is a novel anti-cancer agent treating colorectal cancer because it effectively inhibited tumor growth and induced apoptosis. Our in vitro findings suggested that PARP-1, Caspase-9, p53 and Bcl-2 were involved in CBME-induced apoptosis. These results provide novel insights into the molecular mechanisms underlying the anti-cancer activity of CBME, thereby enabling the development of more efficacious and safer bioactive agents for controlling colorectal cancer.

## Figures and Tables

**Table 1.** Primers used to analyze inflammatory gene expressions in HCT116

Gene	Primer sequence (Forward 5′→3′)	Primer sequence (Reverse 5′→3′)
p53	5′-CTT GCC GTC CCA AGC AAT-3'	5′-TGG CAT TCT GGG AGC TTC AT-3'
Caspase-9	5′-CTT TGT GTC CTA CTC TAC TTT CC-3'	5′-AAC AGC ATT AGC GAC CCT A-3'
p21	5′-CCTCATCCCGTGTTCTCCTTT-3'	5′-GTACCACCCAGCGGACAAGT-3'
Bcl-2	5′-CCA AGA AAG CAG GAA ACC-3'	5′-GGA TAG CAG CAC AGG ATT-3'
β-actin	5′-TCC TCC TGA GCG CAA GTA CTC-3'	5′-CGG ACT CGT CAT ACT CCT GCT T-3'

## Data Availability

The datasets used and/or analyzed during the current study are available from the corresponding author on reasonable requests.
